# Current Diagnosis and Management of Immune Related Adverse Events (irAEs) Induced by Immune Checkpoint Inhibitor Therapy

**DOI:** 10.3389/fphar.2017.00049

**Published:** 2017-02-08

**Authors:** Vivek Kumar, Neha Chaudhary, Mohit Garg, Charalampos S. Floudas, Parita Soni, Abhinav B. Chandra

**Affiliations:** ^1^Department of Medicine, Maimonides Medical CenterBrooklyn, NY, USA; ^2^Department of Pediatrics, Maimonides Medical CenterBrooklyn, NY, USA; ^3^Medical Director, Yuma Regional Cancer CenterYuma, AZ, USA

**Keywords:** immune related adverse events, checkpoint blockade, irAEs, nivolumab, pembrolizumab, ipilimumab

## Abstract

The indications of immune checkpoint inhibitors (ICIs) are set to rise further with the approval of newer agent like atezolimumab for use in patients with advanced stage urothelial carcinoma. More frequent use of ICIs has improved our understanding of their unique side effects, which are known as immune-related adverse events (irAEs). The spectrum of irAEs has expanded beyond more common manifestations such as dermatological, gastrointestinal and endocrine effects to rarer presentations involving nervous, hematopoietic and urinary systems. There are new safety data accumulating on ICIs in patients with previously diagnosed autoimmune conditions. It is challenging for clinicians to continuously update their working knowledge to diagnose and manage these events successfully. If diagnosed timely, the majority of events are completely reversible, and temporary immunosuppression with glucocorticoids, infliximab or other agents is warranted only in the most severe grade illnesses. The same principles of management will possibly apply as newer anti- cytotoxic T lymphocytes-associated antigen 4 (CTLA-4) and programmed cell death protein 1 (PD-1/PD-L1) antibodies are introduced. The current focus of research is for prophylaxis and for biomarkers to predict the onset of these toxicities. In this review we summarize the irAEs of ICIs and emphasize their growing spectrum and their management algorithms, to update oncology practitioners.

## Introduction

Recent advances in cancer immunotherapy are notable for the introduction of a novel class of drugs known as immune checkpoint inhibitors (ICIs). These agents inhibit negative regulatory components of the immune response, such as the cytotoxic T lymphocytes-associated antigen 4 (CTLA-4) and the programmed cell death protein-1 and its ligand (PD-1/PD-L1), which lead to enhanced T cell action against the cancer cells (Hodi et al., 2010). Ipilimumab is an anti- CTLA-4 antibody and was the first agent to receive food and drug administration (FDA) approval for use against advanced-stage melanoma^1^. Since then anti- CTLA-4 antibody tremelimumab, anti-PD-1 antibodies pembrolizumab and nivolumab and PD-L1 antibodies, atezolizumab and durvalumab, have shown beneficial effects in several cancers (Wolchok et al., 2010; Topalian et al., 2012, 2014; Ribas et al., 2013b; Larkin et al., 2015; Robert et al., 2015a; Postow et al., 2015; Fehrenbacher et al., 2016; Ferris et al., 2016; Massard et al., 2016; Reck et al., 2016; Rittmeyer et al., 2017) (Table 1). In contrast to conventional chemotherapy, boosting the immune system leads to a unique constellation of inflammatory toxicities known as immune-related adverse events (irAEs) that may warrant the discontinuation of therapy and/or the administration of immunosuppressive agents (Gangadhar and Vonderheide, 2014; Cousin and Italiano, 2016). The use of these agents is set to increase due to their dramatic impact on survival in a variety of advanced-stage cancers (Horvat et al., 2015). Thus, it is imperative for personnel involved in the care of oncology patients to be well versed with the heterogeneous presentations of irAEs in terms of recognition and management. Here, we review the current literature on appropriate steps in patient evaluation for the prompt diagnosis of irAEs and describe strategies for optimizing patient outcome with suitable treatment.

**Table 1:** **Mechanism of action of immune checkpoint inhibitors and immune-Related Adverse Events^#^**.

	**Ipilimumab (Hodi et al.**, **2010****; Wolchok et al.**, **2010****; Ibrahim et al.**, **2011****; Eggermont et al.**, **2015**, **2016****; Horvat et al.**, **2015****; Larkin et al.**, **2015****; Postow et al.**, **2015****; Robert et al.**, **2015b****)**		**Tremelimumab^@^ (Tarhini et al.**, **2012****; Ribas et al.**, **2013b****; Calabro et al.**, **2015****; Kindler et al.**, **2016****)**		**Nivolumab (Topalian et al.**, **2012**, **2014****; Weber J. S. et al.**, **2013****; Borghaei et al.**, **2015****; Brahmer et al.**, **2015****; Larkin et al.**, **2015****; Rizvi et al.**, **2015****; Robert et al.**, **2015a****; Ferris et al.**, **2016****)**		**Pidilizumab^&^ (Berger et al.**, **2008****; Armand et al.**, **2013****; Westin et al.**, **2014****)**		**Pembrolizumab (Hamid et al.**, **2013****; Garon et al.**, **2015****; Robert et al.**, **2015b****; Herbst et al.**, **2016****; Nanda et al.**, **2016****; Reck et al.**, **2016****; Seiwert et al.**, **2016****)**		**Atezolizumab**^**∧**^ **(Fehrenbacher et al.**, **2016****; Rosenberg et al.**, **2016****; Rittmeyer et al.**, **2017****)**		**Durvalumab^~^ (Massard et al.**, **2016****)**	
Mechanism of action	CTLA-4 inhibitor		CTLA-4 inhibitor		PD-1 inhibitor		PD-1 inhibitor		PD-L1 inhibitor		PD-L1 inhibitor		PD-L1 inhibitor	
Therapeutic Status	FDA approved to reduce the risk of melanoma recurrence after surgery (2015), and late stage melanoma (2011).		Tremelimumab was granted orphan drug status in 2015 for the treatment of malignant mesothelioma but is not FDA approved yet.		FDA approved in Hodgkin's lymphoma (2016), Head and neck cancer (2016), advanced lung cancer (2015), metastatic renal cell carcinoma (2015), advanced melanoma (2014).		Under trial		FDA approved in recurrent or metastatic head and neck cancer (2016), first-line treatment for metastatic non-small cell lung cancer in selected patients (2016), metastatic non-small cell lung cancer (2015), advanced melanoma (2014).		FDA approved in metastatic non-small cell lung cancer (2016) and urothelial carcinoma (2016).		Under trial	
Adverse Events	All	= III	All	= III	All	= III	All	= III	All	= III	All	= III	All	= III
Grades (%)	55–65	10–18	~14	~5	30–45	3–7	None^$^		40–45	7–12	16–21	~ 5	~23	4–5
**SKIN**														
Pruritus	25–30^*^	<1	30–32	1	17	<1			11–21	1	12–14	<1	3–4	0
Rash	33–34^*^	2–3	32–34	2	15	<1	None		10–21	2	15	<1	NR	NR
Vitiligo	3–4	0	NR	NR	10–11	<1			9^*^	<1	NR	NR	NR	NR
**GASTROINTESTINAL**														
Diarrhea	36–38^*^	6–7	}30–40	5–10	8–16	<1	None		8–20	1	18–20	1–2	9–10	0
Colitis	8–10^*^	5–6			1–3	1			1–2	1–2	<1	<1	0	0
**HEPATIC**														
Increased ALT	<1	<1	NR	NR	1–2	<1			2–8^*^	<1	2–3	0		
Increased AST	1–2	<1	NR	NR	1–2	<1	None		3–10^*^	1	2–3	0	None	
Hepatitis	<1	<1	1	1	1–2	1			1–2^*^	<1	1–2	1		
**ENDOCRINE**														
Hypothyroidism	1–2	<1	}5	1	4–5	<1			8–10^*^	<1	2–4	<1		
Hyperthyroidism	0–2	<1			0–3	<1	None		3–4^*^	<1	1	<1	NR	
Hypophysitis	2–3^*^	2–3	2	1	<1	<1			<1	<1	<1	<1		
Pneumonitis	<1	<1	None		1–5	0–2	None		4–6^*^	1–2	2.6	<1	0	0
Renal Failure	1	<1	None		1–3^*^	0–2	None		<1	<1	None		1–2	1
Neurological	<1	<1	None		<1	<1	None		<1	<1	None		NR	

*CTLA-4, cytotoxic T-lymphocyte antigen 4; PD-1, programmed death; PD-L1, programmed death ligand-1; ALT, alanine aminotransferase; AST, aspartate aminotransferase; NR, not reported. Year of FDA approval is written in the bracket*.

* *Current data suggest that these toxicities are more commonly associated with this particular agent*.

$ *Event did not occur*.

# *Adverse events were included only if they were reported as immune related or adverse events of special interest (AESI). Head to head trials to compare toxicities among different agents have not been conducted yet*.

@ *Formerly ticilimumab, CP-675, 206*.

& *Formerly MDV9300. Has been tested in diffuse large B-cell lymphoma and multiple myeloma. Immune related adverse events did not occur in either study. ^∧^Formerly MPDL 3280A*.

~ *Formerly medi4736. Majority of trials on immunotherapy have tested three agents, ipilimumab, nivolumab and pembrolizumab. Data on the toxicities of newer check point inhibitors are still accumulating*.

### Incidence

The incidence of any grade irAEs is reported to range from 15 to 90% (Hodi et al., 2010; Eggermont et al., 2016; Ferris et al., 2016) in single agent trials. The rate of severe irAEs requiring immunosuppression and withdrawal of immunotherapy is estimated to be 0.5–13% (Hodi et al., 2010; Wolchok et al., 2010; Topalian et al., 2012, 2014; Ribas et al., 2013b; Larkin et al., 2015; Postow et al., 2015; Robert et al., 2015a; Ferris et al., 2016; Table S1)^1^. The high incidence reported in a recent study was attributed to subjectivity in toxicity evaluations among investigators and the accumulation of experience leading to early diagnosis (Horvat et al., 2015). The risk of severe grade adverse events increased from 7 to 25% with an increase in the dose of ipilimumab from 3 mg/kg to 10 mg/kg^9^. This was mostly due to increase in the episodes of diarrhea. However, this pattern was not observed when nivolumab dosing was increased from 0.3 mg/kg to 10 mg/kg^10^. Severe grade toxicities with pembrolizumab were also similar at doses of 10 mg/kg every 2 or 3 weeks and its FDA-approved dosage of 2 mg/kg every 3 weeks (Herbst et al., 2016). Thus, it may be argued that toxicities due to anti-CTLA-4 antibodies are dose dependent whereas toxicities with anti-PD-1/anti-PDL-1 antibodies are independent of doses. Ipilimumab, a CTLA-4 inhibitor is associated with higher rates of gastrointestinal (GI) toxicities, pruritus, rash, and hypophysitis whereas use of PD-1/PD-L1 antagonists is associated with higher risk of vitiligo, dysthyroidism, hepatotoxicity, and pneumonitis (Hamid et al., 2013; Weber J. S. et al., 2013; Brahmer et al., 2015; Eggermont et al., 2015, 2016; Herbst et al., 2016; Table 1 and Table S1). The data on toxicities from newer agents continue to accumulate. The irAEs in select clinical trials have been summarized in Table S1.

### Timing

The majority of toxicities appear temporally, with skin manifestations the earliest to appear at 2–3 weeks after the 1st dose of ipilimumab. Immune-mediated colitis and hepatitis appear approximately 5–10 and 12–16 weeks after the 2nd and 3rd dose, respectively. Endocrine dysfunctions present from the 9th week onwards following the 4th dose (Hodi et al., 2010; Ryder et al., 2014). Immune-mediated pneumonitis is seen 8–14 weeks after treatment initiation (Hodi et al., 2010). Immune-mediated nephritis appears much later, after 14–42 weeks on immunotherapy (Izzedine et al., 2014). However, similar temporal association of the appearance of irAEs has not been described for PD-1/PDL-1 antagonists (Naidoo et al., 2015).

### General principles of management

Current guidelines are formulated by manufacturers in collaboration with the FDA and are based on the experience from clinical trials examining the efficacy of ICIs and expert consensus. The guidelines are incorporated in the packaging inserts of these agents^2^^,^^3^^,^^4^^,^^5^. Although anti-PD-1/ anti-PDL-1 antibodies may be less toxic than anti-CTLA-4 antibodies, the approach to managing irAEs due to these agents is similar, with slight variations (Postow and Wolchok, 2016). The severity of adverse events is graded using Common Terminology Criteria for Adverse Events (CTCAE) on a scale from 1 to 5 (1 = mild, 2 = moderate, 3 = severe, 4 = life threatening, and 5 = death related to toxicity) (National Cancer Institute, 2009). However, the grading of irAEs may be challenging, due to arbitrary distinctions between grade 2 and 3 toxicities, such as the number of stools in a day, may be affected by recall bias. Thus, this system of grading may not be entirely suitable to grade ICIs toxicities (Horvat et al., 2015). Therefore, it is prudent to use clinical judgment rather than strictly adhering to the guidelines. We have outlined several general principles that should be followed irrespective of affected organs^2^^,^^3^^,^^4^^,^^5^.

➢ The ICIs are interrupted in moderate (grade 2) irAEs and are resumed when symptoms and/or lab values decrease below grade 1. Glucocorticoids (prednisone 0.5–1 mg/kg/day or equivalent) should be started if symptoms persist beyond 1 week.
➢ For grade 3/4 toxicities, high doses of glucocorticoids (prednisone 1–2 mg/kg/day or equivalent) should be given. The glucocorticoids should be tapered gradually when symptoms subside to grade 1 or less. The eligible patients (those on prednisone 20 mg or equivalent doses for at least 4 weeks) should also receive appropriate prophylaxis against *Pneumocystis jirovecii* as per the established guidelines^6^.
➢ Alternative immunosuppressive agents should be considered (infliximab 5 mg/kg; mycophenolate mofetil in hepatitis) if symptoms continue beyond 3 days on intravenous glucocorticoids. Infliximab 5 mg/kg should be repeated after 2 weeks for persistent symptoms.
➢ For grade 4 toxicities, ICIs should be stopped permanently except in endocrinopathies controlled on hormone replacement. Therapy can be resumed in selected patients with grade 3 toxicities, as discussed in the organ-specific toxicities section.

ICIs should also be stopped permanently in the following circumstances ^2^^,^^3^^,^^4^^,^^5^:

➢ Grade 2 reactions lasting for 6 weeks or longer. However, anti- PD-1/anti-PD-L1 antibodies can be continued in endocrinopathies controlled with hormone replacement.
➢ Inability to reduce glucocorticoids dose to 7.5 mg prednisone or equivalent per day for patients treated with anti-CTLA-4 antibodies and less than 10 mg /day within 12 weeks for anti-PD-1 antibodies.
➢ Grade 2–4 ocular reactions not improving to grade 1 within 2 weeks after treatment with topical immunosuppression or requiring systemic treatment.

### Impact of irAEs and immunosuppression on efficacy

The immunosuppressive agents used to treat irAEs do not appear to affect the response to further immunotherapy (Attia et al., 2005). In contrast to previous studies, a recent retrospective analysis reported similar overall survival in patients who received immunosuppression (Horvat et al., 2015). The association between irAEs and the efficacy of ICIs is also controversial (Attia et al., 2005).

### Biomarkers

Biomarkers which could predict the development of toxicities have been described in the patients on ipilimumab. An increase from baseline in eosinophils and interleukin 17 (IL-17) after treatment has been shown to be associated with irAEs (Callahan et al., 2011; Schindler et al., 2014). On gene profiling, two markers of neutrophil activation, CD177 and CEACAM1 also show promise as biomarkers of ICIs toxicity. These genes are expressed increasingly in the blood of patients, who developed GI toxicity after treatment with anti-CTLA-4 antibodies (Shahabi et al., 2013). Higher risk of GI toxicity was also seen in patients who exhibited evidence of inflammation on colon biopsies like infiltration of lamina propria by neutrophils and presence of cryptic abscesses, erosions and gland destruction prior to the initiation of treatment (Berman et al., 2010). However, routine testing of these biomarkers is not recommended yet.

### Organ-specific immune related adverse events

#### Systemic adverse events

Fatigue is the most common symptom reported by up to 40% of patients after treatment with anti-CTLA-4 antibodies (Weber, 2009; Hodi et al., 2010; Ibrahim et al., 2011; Tarhini et al., 2012; Calabro et al., 2015; Larkin et al., 2015; Kindler et al., 2016) and 16–24% of patients treated with anti-PD-1/anti-PD-L1 antibodies in single-agent trials (Borghaei et al., 2015; Garon et al., 2015; Rizvi et al., 2015; Robert et al., 2015a,b; Nanda et al., 2016; Reck et al., 2016; Rosenberg et al., 2016; Seiwert et al., 2016). This fatigue is usually mild, and the presence of severe fatigue should trigger an assessment for underlying disorders such as endocrinopathies^2^,^3^,^4^,^5^. Infusion reactions, including fever and chills, are more common with CTLA-4 inhibitors accounting for AEs in phase III studies (Momtaz et al., 2015). They are rarely high grade and may be managed supportively with antipyretics and antihistamines (Villadolid and Amin, 2015).

#### Dermatological

Skin manifestations, such as rash/pruritus and mucositis, are the most common irAEs associated with ICIs. Approximately 47–68% of patients treated with anti-CTLA-4 antibodies and 30–40% patients treated with anti-PD-1/anti-PD-L1 antibodies suffer skin toxicities of any grade (Weber, 2009; Hodi et al., 2010; Wolchok et al., 2010; Ibrahim et al., 2011; Tarhini et al., 2012; Topalian et al., 2012; Ribas et al., 2013b; Topalian et al., 2014; Postow et al., 2015; Borghaei et al., 2015; Calabro et al., 2015; Garon et al., 2015; Larkin et al., 2015; Rizvi et al., 2015; Robert et al., 2015a,b; Ferris et al., 2016; Kindler et al., 2016; Nanda et al., 2016; Rosenberg et al., 2016; Seiwert et al., 2016)^1^. The characteristic rash is faintly erythematous and maculopapular, involves the trunk and extremities and may be pruritic (Jaber et al., 2006). Vitiligo is also common and has delayed appearance after several months of treatment with ICIs (Weber, 2012). Histological analysis shows perivascular lymphocytic infiltration deep in the dermis with CD4+ and CD8+ T cells in close proximity with melanocytes (Jaber et al., 2006). The treatment of symptomatic cases with topical glucocorticoids (betamethasone 0.1% cream) or urea-containing creams and oral antipruritic agents (diphenhydramine, hydroxyzine, GABA agonists, or NK-1 receptor antagonists) for troublesome pruritus is usually sufficient (Weber, 2012; Horvat et al., 2015). The majority of skin eruptions are mild, and immunotherapy can be continued in most patients (Weber, 2012). Severe (Grade 3 defined as papules and/or rash covering >30% BSA) cases should be treated with oral glucocorticoids for 3–4 weeks, with temporary discontinuation of ICIs (Table 2). Permanent discontinuation should be considered in more severe cases, such as Stevens–Johnson syndrome, but fortunately severe irAEs are rare. Patients who fail to respond to steroids or have bullae formation merit dermatologic evaluation and skin biopsy. Oral mucositis and dryness are seen more frequently with anti-PD-1/anti-PD-L1 antibodies than with anti-CTLA-4 antibodies (Weber, 2009; Ibrahim et al., 2011; Tarhini et al., 2012; Borghaei et al., 2015; Calabro et al., 2015; Garon et al., 2015; Rizvi et al., 2015; Robert et al., 2015b; Kindler et al., 2016; Nanda et al., 2016; Rosenberg et al., 2016; Seiwert et al., 2016). Topical glucocorticoids and lidocaine are used for treatment. These lesions may mimic oral candidiasis, which should be ruled out^2^^,^^3^^,^^4^^,^^5^.

**Table 2:** **Summary of management of selected immune related adverse events (irAEs)**.

**S.N**.	**Organ system**	**Manifestations**	**Management**
**1**.	**Dermatological** ❖ Most common manifestation and earliest to occur. ❖ Seen in 2–3 weeks after initiation of treatment with ipilimumab. ❖ Commonly seen after the first dose. ❖ Manifests as maculopapular rash, erythema, pruritus, dry skin, alopecia or hypertrichosis, lichenoid keratosis and vitiligo. More common with CTLA-4 inhibitors. Seen in up to 47–68% patient on anti-CTLA-4 therapy and 30–40% patients on anti-PD-1/anti-PD-L1 therapy. Mucositis and vitiligo are more common in patients receiving anti-PD-1/anti-PD-L1 agents	**Immune mediated dermatitis** Grade^*^ 1: Rash affecting <10% BSA. Mostly Asymptomatic	**❖ Diagnosis:** Mucocutaneous examination. **❖ Treatment: Continue immunotherapy** Symptomatic treatment with oral antihistaminic drugs. Topical steroids.
		**Grade 2:** Rash affecting 10–30% BSA.	**❖ Diagnosis:** clinical examination and lab testing for LFTs, KFTs, serum tryptase and IgE levels ❖ Treatment: Withhold immunotherapy^**^. Resume after toxicity improves to grade 1 or lower^@^. Symptomatic treatment with oral antihistaminic drugs. Oral Prednisone 0.5–1 mg/kg/day or equivalent taper over 4 weeks if symptoms resolve.
		**Grade 3/4:** Rash covering >30% BSA. Severe life threatening symptoms Generalized exfoliative/ulcerated rash	**❖ Diagnosis:** As in grade 2 plus skin biopsy **❖ Treatment: Withhold immunotherapy**. Consult Dermatologist. Resume after toxicity improves to grade 1 or lower. Symptomatic treatment with oral antihistaminic drugs. Oral Prednisone^#^ 1–2 mg/kg/day or equivalent taper over 4 weeks if symptoms resolve. Consider alternative immunosuppressive agent (Cyclophosphamide, Mycophenolate mofetil, or Infliximab) if symptoms don't improve after 48 h.
**2**.	**Gastrointestinal** ❖ Seen 5–10 weeks later typically after 2nd dose of ipilimumab. ❖ Manifests as increase in stool frequency, diarrhea or constipation, blood or mucus in stool, abdominal pain/cramping, nausea and vomiting. ❖ More common with anti-CTLA-4 therapy (30–40%). Grade 3 and 4 toxicities 1n 10% patients against 1–2% on anti-PD-1/anti-PD-L1 therapy	**Immune mediated colitis** **Grade 1:** =4 stools/day over baseline. Mild abdominal symptoms	❖ **Diagnosis:** stool microscopic examination for ova, parasites and stool culture. Stool antigen for *c. difficile* if suspected clinically. Lab testing for LFTs and KFTs. ❖ **Treatment: Continue immunotherapy**. Symptomatic treatment and monitor for fluid electrolytes balance. American diet Association (ADA) colitis diet, loperamide or atropine sulfate. Budesonide can be tried if symptoms persist beyond 2–3 days.
		**Grade 2:** Moderate new symptoms. 4–6 stools/day over baseline.	❖ **Diagnosis:** As above in grade 1. Evaluate for other causes like progression of primary disease. Colonoscopy may be beneficial in selected cases. ❖ **Treatment: withhold immunotherapy**. If symptoms improve to grade I resume immunotherapy. Symptomatic treatment. If symptoms are persistent beyond 5–7 days start oral prednisone 1 mg/kg/day or equivalent. Taper over 4 weeks if symptoms improve. Start Infliximab 5 mg/kg every 2 weeks if symptoms don't improve after 3 days on steroid treatment.
		**Grade 3/4:** Severe grade. = 7 stools/day over baseline. Severe and persistent abdominal pain, fever, ileus. Life threatening complications like intestinal perforation and peritonitis	❖ **Diagnosis:** Rule out infectious and other causes as above. GI consult and endoscopy in selected cases ❖ **Treatment: Discontinue immunotherapy** I.V. Methyl prednisone 2–4 mg/kg/day or equivalent taper over 4 weeks if symptoms resolve. Consider alternative immunosuppressive agent (Cyclophosphamide or mycophenolate mofetil) if symptoms don't improve after 48 h. Monitor for intestinal perforation.
**3**.	**Hepatic** ❖ Appears 12–16 weeks after initiation of treatment with ipilimumab. ❖ Typically seen after the 3rd dose of checkpoint inhibitor treatment. ❖ Mostly asymptomatic elevation of liver enzyme. Fever, fatigue and jaundice may be seen in some patients. ❖ Manifests in <10% patients on anti-CTLA-4 therapy but in ~20% patients on combination (anti-CTLA-4 plus anti-PD-1/anti-PD-L1) therapy.	**Immune mediated hepatitis** **Grade 1:** Asymptomatic/mildly symptomatic AST/ALT of 2.5 × ULN Total Bilirubin of 1.5 × ULN	❖ **Diagnosis:** LFTs. ❖ **Treatment: Continue immunotherapy** if asymptomatic. Monitor LFTs until resolution
		**Grade 2:** Symptomatic. AST/ALT of 2.5–5 × ULN Total Bilirubin of 1.5–3 × ULN	❖ **Diagnosis:** Rule out viral, drug induced or autoimmune causes. Monitor LFTs daily till resolution followed by weekly testing. ❖ **Treatment: Withhold immunotherapy**. Resume after toxicity improves to grade 1 or lower. Oral Prednisone 1 mg/kg/day or equivalent taper over 4 weeks if symptoms resolve. Consider alternative immunosuppressive agent (tacrolimus, cyclophosphamide or mycophenolate mofetil) if symptoms don't improve after 48 h. Infliximab is contraindicated due to potential hepatotoxicity.
		**Grade 3/4:** Symptoms as above. AST/ALT of 5 × ULN Total Bilirubin of 3 × ULN	❖ **Diagnosis:** As in grade 2 plus imaging to rule out malignant etiology. Monitor LFTs daily till resolution. ❖ **Treatment: Discontinue immunotherapy** I.V. Methyl prednisone 2–4 mg/kg/day or equivalent taper over 4 weeks if symptoms resolve. If no improvement after 5–7 days, add tacrolimus 0.10–0.15 mg/kg/day (trough level 5–20 ng/mL). Consider alternative agents (cyclophosphamide or mycophenolate mofetil), if no response despite therapeutic levels. Infliximab is contraindicated.
**4**.	**Pulmonary** ❖ Seen after 8–14 weeks of 1st dose of ipilimumab. ❖ Asymptomatic appearance of infiltrates on lung imaging is more common. ❖ Symptomatic pneumonitis is seen in =1%. More common with anti-PD-1/anti-PD-L1 than anti-CTLA-4 therapy	**Immune mediated pneumonitis** **Grade 1:** Asymptomatic, only radiological changes.	❖ **Diagnosis:** Radiological imaging using High resolution computed tomography (HRCT chest). Repeat CT before every cycle ❖ **Treatment: Withhold immunotherapy for 2–4 weeks**. Monitor for symptoms every 3 days. If new symptoms develop, treat as higher grade.
		**Grade 2:** Mild/Moderate new symptoms limiting instrumental activities of daily living.	❖ **Diagnosis:** As above plus microbiological assessment like sputum examination and cultures. ❖ **Treatment: withhold immunotherapy**. If symptoms improve to grade 1 within 72 h resume immunotherapy otherwise **discontinue immunotherapy. Also Discontinue therapy in recurrent grade 2 pneumonitis**. Monitor for symptoms daily. Oral prednisone 1 mg/kg/day or equivalent. Taper over 4 weeks if symptoms improve.
		**Grade 3/4:** Severe symptoms limiting self-care activities of daily living. Hypoxia or Respiratory failure requiring urgent interventions like endotracheal intubation or tracheostomy.	❖ **Diagnosis:** Rule out infectious and other pulmonary causes. Pulmonary consult and bronchoscopy ❖ **Treatment: Discontinue immunotherapy** I.V. Methyl prednisone 2–4 mg/kg/day or equivalent taper over 4 weeks if symptoms resolve. Consider prophylactic antibiotics. Consider alternative immunosuppressive agent (Cyclophosphamide or Infliximab) if symptoms don't improve after 48 h.
**5**.	**Endocrine** ❖ Generally seen 9 weeks after initiation of ipilimumab treatment. ❖ **Immune related thyroiditis** Hypothyroidism/Hyperthyroidism: Fatigue, weakness, asthenia, new onset atrial fibrillation, constipation/diarrhea, cold/heat intolerance, dry skin/excessive diaphoresis, weight gain/weight loss. ❖ **Immune-mediated adrenalitis:** Asthenia, failure to thrive, anorexia, nausea, vomiting, fever, coma, hypotension, hypoglycemia, eosinophilia. ❖ **Immune -mediated hypophysitis:** Headache, visual field defects, blurring of vision, impotence, amenorrhea	**Immune-mediated endocrinopathies** **Grade 1:** Asymptomatic or mild symptoms; clinical or diagnostic observations only; intervention not indicated	❖ **Diagnosis:** Complete blood count, comprehensive metabolic profile. Consult endocrine **Thyroiditis:** TSH. If TSH is below 0.5 × ULN or above 2 × ULN or consistently out of normal range in subsequent cycles consider adding free T3 and T4. **Adrenalitis:** ACTH, Morning serum cortisol-if abnormal Cosyntropin stimulation test. **Hypophysitis:** LH/FSH/Testosterone, Prolactin. MRI brain with pituitary cuts and visual field testing if indicated. ❖ **Treatment: Continue immunotherapy**. Monitor for symptoms. If worsens treat as higher grades. Treat for hyper or hypothyroidism if indicated
	Hypothyroidism is more common with anti-CTLA-4 while hypophysitis and hyperthyroidism is seen more commonly with anti-PD-1/anti-PD-L1 therapy	**Grade 2:** Moderate; minimal, local or noninvasive; intervention indicated; limiting age-appropriate instrumental ADL	❖ **Diagnosis:** As above in grade 1. ❖ **Treatment:** **1. Hyper/Hypothyroidism-continue immunotherapy** **2. Adrenalitis: continue immunotherapy**. **3. Hypophysitis^$^: Withhold immunotherapy**. Prednisone 1–2 mg/kg/day or equivalent. Taper over >4 weeks before resuming immunotherapy. Replace deficient hormone. If patient developed hypophysitis on ipilimumab, it can be replaced with pembrolizumab from next cycle.
		**Grade 3:**Severe or medically significant but not immediately life-threatening; hospitalization or prolongation of existing hospitalization indicated; disabling; limiting ADL and self-care	❖ **Diagnosis:** As above in grade 1. ❖ **Treatment:** **1. Hyper/Hypothyroidism: continue immunotherapy**. Treatment of hypo/hyperthyroidism as per standard guidelines. **2. Adrenal insufficiency: withhold immunotherapy**. Prednisone 1–2 mg/kg/day or equivalent. Taper over >4 weeks before resuming immunotherapy. **3. Hypophysitis: permanently discontinue immunotherapy**. Prednisone 1–2 mg/kg/day or equivalent. Taper over >4 weeks. Few patients may require hormone replacement therapy for life.
		**Grade 4:**Life-threatening consequences; urgent intervention indicated	❖ **Diagnosis:** As above in grade 1. ❖ **Treatment:** **1. Hyper/Hypothyroidism: continue immunotherapy**. Treatment of hypo/hyperthyroidism as per standard guidelines. **2. Adrenal insufficiency: Permanently discontinue immunotherapy**. Prednisone 1–2 mg/kg/day or equivalent. Taper over >4 weeks before resuming immunotherapy. If in adrenal crisis stabilize the patient prior to endocrine work-up. Rule out sepsis. If in shock, start with stress dose steroids, antibiotics and iv fluids. **3. Hypophysitis: permanently discontinue immunotherapy**. Prednisone 1–2 mg/kg/day or equivalent. Taper over >4 weeks. Few patients may require hormone replacement therapy for life.
**6**.	**Renal** ❖ Seen 14–42 weeks after initiation of treatment. ❖ Rare with both anti-CTLA-4 and anti-PD-1/anti-PD-L1 therapy	**Immune mediated Renal dysfunction** **Grade 1:** ↑ creatinine above the baseline but =1.5 ULN	❖ **Diagnosis:** Kidney Function Tests, Urine analysis ❖ **Treatment: Continue immunotherapy**. Symptomatic treatment and monitor for fluid electrolytes balance.
		**Grade 2 and 3:** creatinine 1.5–6 mg/dl ULN	❖ **Diagnosis:** As in grade 1. Monitor creatinine every 2–3 days. Consider renal biopsy. ❖ **Treatment: Withhold immunotherapy**. Oral Prednisone 0.5–1 mg/kg/day or equivalent, if no response ↑ to 1–2 mg/kg/day and discontinue immunotherapy permanently. If elevation persists =7 days treat as grade 4.
		**Grade 4:** creatinine > 6 mg/dl ULN	❖ **Diagnosis:** As in grade 1. Monitor creatinine every day. Consider renal biopsy. Consult nephrology **Treatment: Withhold immunotherapy**. Oral Prednisone 1–2 mg/kg/day. Taper at least over 4 weeks.
**7**.	**Neurologic** Headache, fever, stiffness, memory problem, confusion, drowsiness, hallucinations, seizures, peripheral neuropathy	**Immune-mediated neurological adverse reactions** **Grade 1:** Asymptomatic or mildly symptomatic	❖ **Diagnosis:** Clinical examination ❖ **Treatment: Continue immunotherapy**. Monitor for progression of disease.
	Rare with both anti-CTLA-4 and anti-PD-1/anti-PD-L1 therapy	**Grade 2:** New onset moderate symptoms limiting instrumental activities of daily living.	❖ **Diagnosis:** Monitor for progression of disease. ❖ **Treatment: Withhold immunotherapy**. Consider consulting neurology. Oral prednisone 0.5–1 mg/kg/day or equivalent. If no response treat as grade 3 and 4.
		**Grade 3 and 4:** New onset severe symptoms affecting self-care activities of daily living. Life threatening.	❖ **Diagnosis:** MRI brain, lumbar puncture, nerve conduction velocity, electromyography, skin, nerve or muscle biopsy as clinically indicated. ❖ **Treatment: Permanently discontinue immunotherapy**. Consult neurology. Prednisone 1–2 mg/kg/day or equivalent. Taper over at least 4 weeks. If worsens or atypical presentation consider other immunosuppressive agents.

*ADL, activities of daily living; ALT, alanine aminotransferase; AST, aspartate aminotransferase; BSA, body surface area; CTLA-4, cytotoxic T-lymphocyte antigen-4; FSH, follicular stimulating hormone; KFT, kidney function test; LFT, liver function tests; LH, luteinizing hormone; MRI, magnetic resonance imaging; PD-1, programmed cell death; PD-L1, programmed cell death ligand-1; TSH, thyroid stimulating hormone; ULN, upper limit of normal*.

*For References see text*.

* *Common Terminating Criteria for Adverse Events Grading*.

# *Patients receiving prednisone = 20 mg/day or equivalent doses for at least 4 weeks are candidates for pneumocystis jirovecii prophylaxis as per National Comprehensive Cancer Network NCCN guidelines*.

** *Treatment with checkpoint inhibitors is permanently discontinued in grade 2 toxicity if it persists beyond 6 weeks. However PD-1 inhibitors can be continued with hormone replacement in endocrinopathies*.

@ *Nivolumab is permanently discontinued if prednisone can't be tapered to <7.5 mg/day or equivalent without recurrence of symptoms and ipilimumab is discontinued if prednisone can't be tapered below 10 mg/kg/day or equivalent dose*.

$ *Prednisone dose in hypophysitis is controversial. New data suggests that physiological dose is as effective as higher doses advocated previously. See text*.

#### Gastrointestinal

Diarrhea and colitis are more common with anti-CTLA-4 antibodies and are reported in 30–40% of patients treated with ipilimumab (Hodi et al., 2010). Grade 3/4 diarrhea is seen in up to 10% of patients on ipilimumab therapy and 1–2% of cases treated with anti-PD-1/anti-PD-L1 antibodies alone (Weber, 2009; Ibrahim et al., 2011; Tarhini et al., 2012; Borghaei et al., 2015; Calabro et al., 2015; Garon et al., 2015; Rizvi et al., 2015; Robert et al., 2015b; Kindler et al., 2016; Nanda et al., 2016; Rosenberg et al., 2016; Seiwert et al., 2016). Enteritis without colonic involvement leading to small bowel obstruction can also be seen. Colitis predominantly affects the descending colon (Oble et al., 2008). Patients with significant diarrhea/colitis during ipilimumab treatment have subsequently been treated with anti-PD-1/anti-PD-L1 antibodies without developing diarrhea/colitis (Naidoo et al., 2015).

Grade 1 gastrointestinal events are classified as increase in stool frequency of less than 4 per day or mildly increased ostomy output from baseline (Table 2). Mild cases should be managed symptomatically with loperamide, oral hydration and electrolyte replacement (Weber et al., 2015). Other etiologies such as infection with *Clostridium difficile* or other viral/bacterial pathogens should be excluded (Du-Thanh et al., 2015). The American Dietary Association colitis diet may also be beneficial^2^,^3^,^4^,^5^. Treatment with glucocorticoids is indicated for worsening of symptoms or persistence beyond 3 days. Grade 2 severity events are 4–6 stools per day over baseline or a moderate increase in ostomy output. These events are managed by oral diphenoxylate, atropine or budesonide, in addition to symptomatic treatment. It is important to rule out colitis by performing a colonoscopy in persistent grade 2 or grade 1 diarrhea with hematochezia. Grade 2 diarrhea with bleeding/ulceration should be treated with oral glucocorticoids and interruption of immunotherapy. Grade 3 events include diarrhea with 7 or more stools per day above the baseline, incontinence or severe increase in ostomy limiting self-care activities. Grade 4 is any life-threatening complication, such as bowel perforation, requiring acute intervention. Grade 3 and 4 events should be treated with intravenous glucocorticoids in addition to symptomatic treatment^2^^,^^3^^,^^4^^,^^5^.

The symptomatic improvement should be noted in 48–72 h. Hospitalization is warranted for parenteral glucocorticoids with fluid and electrolyte management in cases refractory to oral glucocorticoids (Weber, 2012; Weber et al., 2015). If the symptoms fail to resolve after 3 days on intravenous glucocorticoids (methylprednisolone 2 mg/kg or equivalent), then infliximab should be given at a dose of 5 mg/kg every 2 weeks^2^,^3^,^4^,^5^. The use of infliximab for this indication is based on its efficacy in Crohn's disease (Minor et al., 2009). However, the recommendations are not clear for non-resolving symptoms on infliximab therapy, and prolonged courses of glucocorticoids are preferred. These patients should be cautiously observed for GI complications, including perforation and obstruction. The prompt intervention is mandatory because colitis related mortality has been attributed to delayed reporting and lack of ICI withholding (Naidoo et al., 2015). The efficacy of matrix budesonide for prophylaxis against colitis has not been proven and is not recommended (Weber et al., 2009).

#### Hepatic

Hepatotoxicity can be caused by both anti-CTLA-4 and anti-PD-1/anti-PD-L1 antibodies. Hepatotoxicity occurs in 2–9% of patients (Weber, 2009; Ibrahim et al., 2011; Tarhini et al., 2012; Borghaei et al., 2015; Calabro et al., 2015; Garon et al., 2015; Rizvi et al., 2015; Robert et al., 2015b; Kindler et al., 2016; Nanda et al., 2016; Rosenberg et al., 2016; Seiwert et al., 2016). Liver function should be tested at baseline and prior to each cycle of immunotherapy. The patients should also be monitored regularly during the post-treatment period. An asymptomatic elevation of hepatic transaminases and hyperbilirubinemia is common, and concomitant fever can also occur (Weber, 2012). Liver biopsy is reserved for unclear cases and reveals prominent sinusoidal histiocytic infiltrates and central vein damage with endotheliitis suggestive of ipilimumab-associated hepatitis (Johncilla et al., 2015). Grade 2 reactions require interruption of cancer treatment, with daily or alternate-day monitoring of liver enzymes until they decrease, and then subsequent weekly assessments (Table 2). Grade 3 or greater irAEs involve AST/ALT levels >5 times upper limit of normal (ULN) or bilirubin >3 times ULN. Severe hepatotoxicity requires high-dose intravenous glucocorticoids for 24–48 h followed by a slow taper for the next 30 days. The glucocorticoids should be switched to mycophenolate 500 mg every 12 h if the liver enzymes are still elevated after 48 h of treatment^2^,^3^,^4^,^5^. The use of infliximab is contraindicated due its potential hepatotoxicity. The differential diagnoses of immunotherapy-induced liver damage include metastasis to the liver, viral hepatitis and other drug toxicity meriting extensive analysis. Hepatitis persisting for longer periods requires prolonged or repeated glucocorticoids tapering (≥4 weeks) and/or additional immunosuppression^2^^,^^3^^,^^4^^,^^5^.

#### Endocrine

Endocrinopathies can occur secondary to inflammation of the pituitary, thyroid and adrenal glands or may be related to development of type-1 diabetes mellitus. Clinical presentation is confounded by nonspecific symptoms such as behavioral changes, nausea, headache, fatigue and visual complaints (Corsello et al., 2013). Hypophysitis and hypothyroidism are the most common endocrinopathies seen in up to 10% of patients treated with anti-CTLA-4 and anti-PD-1/anti-PD-L1 antibodies (Weber, 2009; Hodi et al., 2010; Wolchok et al., 2010; Ibrahim et al., 2011; Topalian et al., 2012, 2014; Tarhini et al., 2012; Ribas et al., 2013b; Borghaei et al., 2015; Calabro et al., 2015; Garon et al., 2015; Larkin et al., 2015; Postow et al., 2015; Rizvi et al., 2015; Robert et al., 2015a,b; Ferris et al., 2016; Kindler et al., 2016; Nanda et al., 2016; Seiwert et al., 2016; Rosenberg et al., 2016)^1^.

Hypophysitis with pituitary dysfunction requires testing for thyroid stimulating hormone (TSH), serum cortisol, adrenocorticotropic hormone (ACTH), growth hormone (GH), prolactin, luteinizing hormone (LH), and follicular stimulating hormone (FSH) in women or testosterone levels in men. Diagnosis is based on clinical symptoms with radiographic abnormalities (pituitary enlargement with enhancement) and biochemical test results (low tropic hormones)^2^^,^^3^^,^^4^^,^^5^. The role of high dose glucocorticoids (1 mg/kg prednisone daily) is controversial in cases with suspected hypophysitis. The use of physiological replacement doses has been suggested, and high doses should be reserved for patients with symptoms related to mass effects such as severe headaches or visual disturbances (Albarel et al., 2015). A recent study reported that TSH and FSH normalized after a follow-up period of 33 months. However, ACTH remained low, with persistent pituitary abnormalities on MRI irrespective of glucocorticoid dose (Albarel et al., 2015; Min et al., 2015).

The routine monitoring of thyroid function is indicated before each dose of ipilimumab. Hypothyroidism is more common than hyperthyroidism. It is important to distinguish primary hypothyroidism (low free T4 with high TSH) from secondary disease (low free T4 with low TSH) caused by hypophysitis. The treatment of hypo and hyperthyroidism should be consistent with standard guidelines (Table 2)^2^^,^^3^^,^^4^^,^^5^. Immunotherapy can be continued with hormone replacement.

Hypophysitis^2^^,^^3^^,^^4^^,^^5^ with clinically significant adrenal insufficiency (hypotension, dehydration, and dyselectrolytemia) is equivalent to adrenal crisis and is a medical emergency that mandates hospitalization, evaluation by an endocrinologist and treatment with methylprednisolone. It is important to distinguish this condition from sepsis, and prompt testing with cultures is mandatory (Table 2).

#### Pulmonary

Grade 3 or higher pneumonitis has been reported in 5–7% of NSCLC patients treated with nivolumab and pembrolizumab (Langer, 2015; Abdel-Rahman and Fouad, 2016). The incidence of symptomatic pneumonitis is only 1% with ipilimumab (Barjaktarevic et al., 2013). The risk increased in patients with prior thoracic radiation. There have been reported granulomatous reactions similar to sarcoidosis (Berthod et al., 2012). The presence of infiltrates on chest radiographs or CT imaging is more common and resolves rapidly after withholding the drug. Pneumonitis should be excluded by CT imaging in any patient with cough, shortness of breath, and fever^2^^,^^3^^,^^4^^,^^5^. A bronchoscopy may reveal diffuse lymphocytic infiltration and should be performed in moderate to severe cases to exclude infections. Severe cases should be treated with glucocorticoids using 2 mg/kg intravenous methyl prednisolone. Immunotherapy should be permanently discontinued in cases with recurrent grade 2–4 irAEs (Table 2)^2^^,^^3^^,^^4^^,^^5^.

### Rare events

#### Ocular

Common ocular manifestations include episcleritis, conjunctivitis and uveitis. The incidence of these events is higher with ipilimumab but remains less than 1% (Huillard et al., 2014; Abu Samra et al., 2016). The patient should be referred to an ophthalmologist and treatment with topical glucocorticoids is required in most cases. The use of oral glucocorticoid therapy is reserved for severe events.

#### Renal

ICIs can cause acute kidney injury that presents similar to other drug-induced tubulointerstitial nephritis. The median duration for the appearance of the kidney injury is 13 weeks (Cortazar et al., 2016). In addition to nephritis, granulomatous lesions and thrombotic microangiopathy can also be seen on renal biopsy. A previous study demonstrated that renal function partially improved after glucocorticoid treatment, and that one-third of patients required dialysis (Cortazar et al., 2016). Grade 2 or higher toxicity is treated with glucocorticoids (Table 2). The immunotherapy should be withheld for Grade 2–3 events and permanently discontinued for Grade 4 events or resistant Grade 2–3 irAEs^2^^,^^3^^,^^4^^,^^5^.

#### Pancreatic

Routine monitoring of amylase/lipase in otherwise asymptomatic individuals is not recommended. Asymptomatic elevation does not require treatment. The significance of elevated amylase and lipase in a large number of patients remains unclear (Ribas et al., 2013b; Postow et al., 2015; Herbst et al., 2016).

#### Neurological

The reported neurologic complications of immunotherapies include posterior reversible encephalopathy syndrome (Maur et al., 2012), Guillain-Barre Syndrome (Wilgenhof and Neyns, 2011), myasthenia gravis (Liao et al., 2014), transverse myelitis (Liao et al., 2014), and neuropathy (de Maleissye et al., 2016). Serious cases should be treated with glucocorticoids and a neurologist should be consulted for additional therapies such as intravenous immunoglobulin and plasmapheresis. (for detailed management see Table 2)

#### Hematological

Autoimmune anemia (Kong et al., 2016), neutropenia (Akhtari et al., 2009), thrombocytopenia (Ahmad et al., 2012), and acquired hemophilia A have been reported (Delyon et al., 2011). Symptom management is similar to other irAEs and involves the use of glucocorticoids and alternative immunosuppression in refractory cases.

### Combination therapy

The distinct mechanisms of action of anti-CTLA-4 and anti-PD-1/anti-PD-L1 antibodies have led to trials examining combination therapies in a variety of malignancies. The incidence of severe adverse events due to the combination of ipilimumab and nivolumab is reported to be 55%, which is significantly higher than either agent individually and leads to discontinuation of treatment in one-third of patients (Larkin et al., 2015). The toxicity profile of ipilimumab varies with the chemotherapy agent used in combination (Weber J. et al., 2013). The combination with dacarbazine is more hepatotoxic (Robert et al., 2011), and carboplatin and taxanes treatment leads to more cutaneous manifestations (Arriola et al., 2016). There are more nephrotoxic and hepatotoxic events observed when combined with vemurafenib (Ribas et al., 2013a). The combination of ipilimumab (dose 10 mg/kg) with granulocyte-macrophage colony-stimulating factor showed fewer gastrointestinal and pulmonary irAEs than ipilimumab alone (45 vs. 58%)(Hodi et al., 2014). However, the efficacy of this combination is not clear at the FDA-approved dose of 3 mg/kg and requires further confirmation.

### Autoimmune conditions

The safety of ICIs in patients with preexisting autoimmune diseases is not clear, and there is a theoretical concern regarding the exacerbation of preexisting conditions. There have been anecdotal reports of patients with anti-muscle antibodies developing rhabdomyolysis with polymyositis (Bilen et al., 2016), and neurotoxicity has been described in patients with anti-neuronal antibodies (Williams et al., 2016). Ipilimumab treatment led to the exacerbation of previously diagnosed autoimmune conditions in 27% of patients within 6 weeks of treatment, and these conditions were easily managed with steroids (Johnson et al., 2015). Clinicians should engage patients in discussions for trial of these agents due to the significant benefit of these antibodies in life-threatening malignancies. The development of new autoimmune syndromes (30% sicca syndromes, 70% inflammatory arthritis and 40% ANA positivity) have also been reported in patients without any prior history of rheumatic conditions (Cappelli et al., 2017).

## Conclusion

ICIs targeting CTLA-4 and PD-1/PDL-1 have dramatically changed the outcomes of patients with many advanced-stage malignancies. However, their introduction is associated with unique irAEs that are mostly transient and mild but can occasionally be fatal. Rapid identification and appropriate treatment can improve outcomes without compromising the efficacy of these agents. In the absence of prospective data, these patients should be managed as per established guidelines based upon pooled clinical experience. Additional data on toxicities will enable us to utilize the full therapeutic potential of these novel drugs.

## Author contributions

VK: original idea, reviewed literature and manuscript writing and editing. AC: mentored, writing and editing of manuscript. NC: reviewed literature and manuscript writing and editing. MG: writing and editing of manuscript. CF: editing and proofreading. PS: editing and proofreading.

### Conflict of interest statement

The authors declare that the research was conducted in the absence of any commercial or financial relationships that could be construed as a potential conflict of interest.

## Footnotes

^1^*Drugs@FDA: FDA Approved Drug Products*. Available online at www.Accessdata.fda.gov/scripts/cder/drugsatfda/index.cf.

^2^http://www.accessdata.fda.gov/drugsatfda_docs/label/2016/761034s000lbl.pdf

^3^Yervoy® [package insert]. Princeton, NJ: Bristol-Myers Squibb Company, 2016. Available online: http://dailymed.nlm.nih.gov/dailymed/drugInfo.cfm?setid=2265ef30-253e-11df-8a39-0800200c9a66

^4^Keytruda® [package insert]. Whitehouse Station, NJ: Merck, and Co., Inc., 2016. Available online: http://dailymed.nlm.nih.gov/dailymed/drugInfo.cfm?setid=9333c79b-d487-4538-a9f0-71b91a02b287

^5^Opdivo® [package insert]. Princeton, NJ: Bristol-Myers Squibb Company, 2016. Available online: http://dailymed.nlm.nih.gov/dailymed/drugInfo.cfm?setid=f570b9c4-6846-4de2-abfa-4d0a4ae4e394

^6^http://www.nccn.org/professionals/physician_gls/pdf/infections.pdf.
