# Corrigendum: Current Diagnosis and Management of Immune Related Adverse Events (irAEs) Induced by Immune Checkpoint Inhibitor Therapy

**DOI:** 10.3389/fphar.2017.00311

**Published:** 2017-05-31

**Authors:** Vivek Kumar, Neha Chaudhary, Mohit Garg, Charalampos S. Floudas, Parita Soni, Abhinav B. Chandra

**Affiliations:** ^1^Department of Medicine, Maimonides Medical CenterBrooklyn, NY, USA; ^2^Department of Pediatrics, Maimonides Medical CenterBrooklyn, NY, USA; ^3^Medical Director, Yuma Regional Cancer CenterYuma, AZ, USA

**Keywords:** check point inhibitors, immune related adverse events, nivoulmab, pembrolizumab, ipilimumab

## Error in Table 1

The Therapeutic Status for Tremelimumab in Table 1 was incorrect. In the original article it was: FDA approved in malignant mesothelioma (2015)

Corrected: Tremelimumab was granted orphan drug status in 2015 for the treatment of malignant mesothelioma but is not FDA approved yet.

## Error in Abstract

In the original article, there was an error: The indications of immune checkpoint inhibitors (ICIs) are set to rise further with the approval of newer agents like tremelimumab and atezolimumab for use in patients with advanced stage mesothelioma and urothelial carcinoma respectively.

Corrected sentence: The indications of immune checkpoint inhibitors (ICIs) are set to rise further with the approval of newer agent like atezolimumab for use in patients with advanced stage urothelial carcinoma.

## Errors in Supplementary Table S1

Original Article: Ribas et al. on Tremelimumab in grade >3 toxicities Endocrine adverse effects is 6(2)

Corrected: Not reported (NR)
Original Article: Massard et al. on Durvalumab in grade >3 toxicities Diarrhea is NR
Corrected: 0 (0).

The authors apologize for these errors and state that this does not change the scientific conclusions of the article in any way. The original article and supplementary material has been updated with these corrections.

### Conflict of interest statement

The authors declare that the research was conducted in the absence of any commercial or financial relationships that could be construed as a potential conflict of interest.

