# Supplementary material for: Current Diagnosis and Management of Immune Related Adverse Events (irAEs) Induced by Immune Checkpoint Inhibitor Therapy
Source: Front Pharmacol. 2017 Feb 8;8:49. doi: 10.3389/fphar.2017.00049 (PMC5296331; doi:10.3389/fphar.2017.00049)
Supplement: Supplementary file 1 [file Table1.pdf]

Table S1. Immune related adverse events (irAEs) in select clinical trials.

Clinical Trials	Details	Number of patients, type of Immune Checkpoint Inhibitor and dose.	Adverse events		
			Organ system / event	All grade	Grade ≥ 3
**Anti- cytotoxic T-lymphocyte –associated protein 4 (CTLA 4)**					
**Eggermont et al.**[19]<br>2016<br>Ipilimumab	Phase 3<br>stage III<br>melanoma	471 patients on ipilimumab at dose of 10ml/kg every 3 weeks for 4 doses then every 3 monthly for up to 3 years (reported after median follow up of 5.3 years)	Any irAEs<br>Dermatological<br>Rash<br>Gastrointestinal<br>Diarrhea<br>Colitis<br>Endocrine<br>Hypophysitis<br>Hepatic<br>↑ Liver enzymes<br>Neurologic	426 (90.4)<br>298(63.3)<br>161(34.2)<br>217(46)<br>194(41)<br>73(15.5)<br>178(37.8)<br>77(16.3)<br>115(24.2)<br>83(17.6)<br>21(4.5)	201 (42.7)<br>20(4.2)<br>5(1.1)<br>70(16.1)<br>46(9.8)<br>39(8.2)<br>37(7.9)<br>21(4.4)<br>51(10.8)<br>20(4.3)<br>9(1.9)
**Eggermont et al.**[20]<br>2015<br>Ipilimumab	Phase 3<br>Stage III<br>melanoma	471 patients on ipilimumab at dose of 10mg/kg every 3 weeks for 4 doses then every 3 monthly for up to 3 years (reported after median follow up of 2.7 years)	Any irAEs<br>Dermatologic<br>Pruritus<br>Rash<br>Gastrointestinal<br>Diarrhea<br>Colitis<br>Endocrine<br>Hypophysitis<br>Hypothyroidism<br>Hepatic<br>↑ Liver enzymes<br>Neurologic	226(48)<br>277(59)<br>176(37)<br>156(33)<br>142(30)<br>150(32)<br>39(8)<br>137(29)<br>62(13)<br>41(9)<br>68(14)<br>68(14)<br>12(3)	200(43)<br>21(4)<br>2(11)<br>6(17)<br>75(16)<br>45(10)<br>36(8)<br>40(9)<br>24(6)<br>1(<1)<br>50(11)<br>25(5)<br>9(2)
**Hodi et al.**[1]<br>2010<br>Ipilimumab	Phase 3<br>Unresectable<br>stage III / IV<br>melanoma	137 patients on ipilimumab alone at dose of 3mg/kg every 3 weeks up to 4 doses.	Any irAEs<br>Dermatologic<br>Pruritus<br>Rash<br>Gastrointestinal<br>Diarrhea<br>Colitis<br>Endocrine<br>Hypothyroidism<br>Hypophysitis<br>Adrenal insufficiency<br>Hepatic	80(61.1)<br>57(43.5)<br>32(2.4)<br>25(19.1)<br>38(29)<br>36(27.5)<br>10(7.6)<br>10(7.6)<br>2(1.5)<br>3(2.3)<br>2(1.5)<br>5(3.8)	19(14.5)<br>2(1.5)<br>0 (0)<br>1(<1)<br>10(7.6)<br>6(4.6)<br>7(5.3)<br>5(3.8)<br>0 (0)<br>2(1.6)<br>2(1.5)<br>0 (0)

			↑ ALT<br>↑ AST<br>Hepatitis	2(1.5)<br>1(<1)<br>1(<1)	0 (0)<br>0 (0)<br>0 (0)
**Wolchok et al.**[9]<br>2010<br>Ipilimumab	Phase 2<br>Previously treated stage III / IV melanoma	71 patients on ipilimumab at dose of 10mg/kg every 3 weeks for 4 doses followed by maintenance every 3 months.	Any irAEs<br>Dermatological<br>Gastrointestinal<br>Endocrine<br>Hepatic	50 (68)<br>33(45)<br>28(38)<br>3(4)<br>2(3)	18(25)<br>3(4)<br>11(15)<br>1(1)<br>2(3)
		71 patients on ipilimumab at dose of 3 mg/kg every 3weeks for 4 doses followed by maintenance every 3 months	Any irAEs<br>Dermatological<br>Gastrointestinal<br>Endocrine<br>Hepatic	46(64)<br>32(44)<br>23(32)<br>4(6)<br>0 (0)	5(7)<br>1(1)<br>2(3)<br>2(3)<br>0 (0)
		71 patients on ipilimumab at dose of 0.3 mg/kg every 3weeks for 4 doses followed by maintenance every 3 months	Any irAEs<br>Dermatological<br>Gastrointestinal<br>Endocrine<br>Hepatic	19(26)<br>09(13)<br>12(17)<br>0 (0)<br>0 (0)	0 (0)<br>0 (0)<br>0 (0)<br>0 (0)<br>0 (0)
**Postow et al.**[8]<br>2015<br>Ipilimumab	Phase 2<br>Unresectable, previously untreated, stage III and IV melanoma	46 patients on ipilimumab at dose of 3 mg/kg.	Dermatological<br>Gastrointestinal<br>Diarrhea<br>Colitis<br>Endocrine<br>Hypothyroidism<br>Hypophysitis<br>Adrenal insufficiency<br>Hepatic<br>↑ ALT<br>↑ AST<br>↑ Lipase<br>Pneumonitis<br>Discontinuation of the therapy	27 (58)<br>17 (37)<br>17(37)<br>6 (13)<br>8 (17)<br>7 (15)<br>3 (7)<br>2 (4)<br>2 (4)<br>2(4)<br>2(4)<br>2(4)<br>2 (4)<br>8(17)	0 (0)<br>5 (11)<br>5(11)<br>3 (7)<br>2 (4)<br>0 (0)<br>2 (4)<br>1 (2)<br>0 (0)<br>0 (0)<br>0 (0)<br>0 (0)<br>1 (2)<br>6(13)
**Larkin et al.**[6]<br>2015<br>Ipilimumab	Phase 3<br>Stage III or IV melanoma	311 patients on ipilimumab alone at dose of mg/kg every weeks	Any IrAEs<br>Dermatological<br>Pruritus<br>Rash<br>Vitiligo<br>Gastrointestinal<br>Diarrhea<br>Colitis<br>Endocrine	229 (73.6)<br>168 (54)<br>110 (35.4)<br>65 (20.9)<br>12 (3.9)<br>114 (36.7)<br>103 (33.1)<br>36 (11.6)<br>34 (10.9)	58 (18.6)<br>9 (2.9)<br>1 (0.3)<br>5 (1.6)<br>0 (0)<br>36 (11.6)<br>19 (6.1)<br>27 (8.7)<br>7 (2.3)

			Hypothyroidism	27 (8.6)	0 (0)
			Hyperthyroidism	13 (4.2)	0 (0)
			Hypophysitis	12 (3.9)	6 (1.9)
			Hepatic	22 (7.1)	5 (1.6)
			↑ ALT	12(3.9)	5(1.6)
			↑ AST	11(3.5)	2 (0.6)
			Pulmonary	6 (1.9)	1 (0.3)
			Pneumonitis	5 (1.6)	1 (0.3)
**Ribas et al.[3]**	Phase 3	325 patients on tremelimumab	Endocrine	24 (7)	NR
2013	Stage IIIc/ IV	at dose of 15mg/kg every 90	Thyroid	17 (5)	2(1)
Tremelimumab	melanoma	days	Hypothalamus and pituitary	6 (2)	4(1)
			Adrenal insufficiency	4(1)	3(1)
			Pancreatitis	3(1)	3(1)
			Hepatitis	2(1)	2(1)
**Anti- programmed cell death protein-1/-Ligand1 (PD-1/PD-L1)**					
**Borghaei et al.[44]**	Phase 3	292 patients on nivolumab at	Rash	27(9)	1(<1)
2015	IIIb/IV or	dose of 3mg/kg every 2 weeks.	Pruritus	24(8)	0 (0)
Nivolumab	recurrent		Diarrhea	22(8)	2(1)
	nonsquamous		Colitis	2(1)	1(<1)
	NSCLC		Hypothyroidism	19(7)	0 (0)
			Hyperthyroidism	4(1)	0 (0)
			↑ ALT	9(3)	0 (0)
			↑ AST	9(3)	1(<1)
			Pneumonitis	8(3)	3(1)
			ILD	2(1)	1(<1)
			↑ Creatinine	5(2)	0 (0)
			Renal Failure	1(<1)	0 (0)
**Topalian et al.[10]**	Phase 1	107 patients on nivolumab at	Any irAEs	58 (54.2)	5 (4.7)
2014	Advanced	dose of 1mg/kg or 3mg/kg or	Dermatological	38 (35.5)	0 (0)
Nivolumab	melanoma	10mg/kg once every 2 weeks	Rash	25(23.4)	0 (0)
		for up to 96 weeks.	Pruritus	14(3.1)	0 (0)
			Gastrointestinal	19 (17.8)	2 (1.9)
			Diarrhea	19 (17.8)	2 (1.9)
			Colitis	2(2)	1 (0.9)
			Hypothyroidism	6(5.6)	1 (0.9)
			Hyperthyroidism	2(1.9)	0 (0)
			Hepatic	7(6.5)	1 (0.9)
			↑ ALT	5(4.7)	0 (0)
			↑ AST	4(3.7)	0 (0)
			Pneumonitis	2(2)	0 (0)
			Renal Failure	2(2)	0 (0)

			Infusion Reactions	6(5.6)	0 (0)
**Rizvi et al.[46]**<br>2015<br>Nivolumab	Phase2<br>squamous<br>NSCLC	117 patients on nivolumab at dose of 3mg/kg every 2 weeks	Dermatological<br>Rash<br>Diarrhea<br>Endocrine<br>Hypothyroidism<br>Hyperthyroidism<br>Pneumonitis<br>Renal Failure<br>↑ ALT	18(15)<br>13(11)<br>12(10)<br>7(6)<br>3(3)<br>1(1)<br>6(5)<br>1(1)<br>1(1)	2(2)<br>1(1)<br>3(3)<br>1(1)<br>0 (0)<br>0 (0)<br>4(3)<br>0 (0)<br>0 (0)
**Larkin et al.[6]**<br>2015<br>Nivolumab	Phase 3<br>Stage III or IV<br>melanoma	313 patients on nivolumab at dose of 3mg/kg every 2 weeks	Any irAEs<br>Dermatological<br>Pruritus<br>Rash<br>Vitiligo<br>Gastrointestinal<br>Diarrhea<br>Colitis<br>Endocrine<br>Hypothyroidism<br>Hyperthyroidism<br>Hypophysitis<br>Hepatic<br>↑ ALT<br>↑ AST<br>Pulmonary<br>Pneumonitis	194 (62)<br>131 (41.9)<br>59(18.8)<br>81(25.9)<br>23 (7.3)<br>61 (19.5)<br>60 (19.2)<br>4 (1.3)<br>45 (14.4)<br>27(8.6)<br>4(1.3)<br>2(0.6)<br>20 (6.4)<br>12(3.8)<br>12(3.8)<br>5 (1.6)<br>4 (1.3)	24 (7.7)<br>5 (1.6)<br>0 (0)<br>2(0.6)<br>1 (0.3)<br>7 (2.2)<br>7(2.2)<br>2 (0.6)<br>2 (0.6)<br>0 (0)<br>2 (0.6)<br>1 (0.3)<br>8 (2.6)<br>4(1.3)<br>3(1)<br>1 (1.3)<br>1 (0.3)
**Robert et al.[5]**<br>2016<br>Nivolumab	Phase 3<br>recurrent<br>squamous cell<br>carcinoma of<br>head and<br>neck	240 patients on nivolumab at dose of 3mg/kg every 2 weeks	Dermatological<br>Rash<br>pruritus<br>Gastrointestinal<br>Diarrhea<br>Endocrine<br>Hypothyroidism<br>Hyperthyroidism<br>Hepatic<br>↑ ALT<br>↑ AST<br>Pneumonitis<br>Renal Failure	37(15.7)<br>18(7.6)<br>17(7.2)<br>16(6.8)<br>16(6.8)<br>18 (7.6)<br>9(3.8)<br>2 (0.8)<br>5 (2.1)<br>2 (0.8)<br>2 (0.8)<br>5(2.1)<br>1(0.4)	0(0)<br>0(0)<br>0(0)<br>0(0)<br>0(0)<br>1(<1)<br>0(0)<br>0(0)<br>2 (0.8)<br>1 (0.4)<br>1 (0.4)<br>2(0.8)<br>0(0)
**Martin et al.[11]**<br>2016	Stage IV<br>NSCLC	154 patients on pembrolizumab at dose of	Any irAEs<br>Sever skin rash	45(29.2)<br>6(3.9)	15(9.7)<br>6(3.9)

Pembrolizumab		200mg every 3 weeks	Colitis	3(1.9)	2(1.3)
			Hypothyroidism	14(9.1)	0 (0)
			Hyperthyroidism	12(7.8)	0 (0)
			Thyroiditis	4(2.6)	0 (0)
			Hypophysitis	1(6.6)	1(6.6)
			Myositis	3(1.9)	0 (0)
			Nephritis	1(0.6)	1(0.6)
			Pneumonitis	9(5.8)	4(2.6)
			Infusion reaction	7(4.5)	0 (0)
			Pancreatitis	1(0.6)	1(0.6)
			T1DM	1(0.6)	1(0.6)
**Seiwert et al.**[47]<br>2016<br>Pembrolizumab	Phase 1b<br>Recurrent or metastatic squamous cell carcinoma of the head and neck	60 patients on pembrolizumab at dose of<br>10mg/kg every 2 weeks	Any irAEs	9 (15)	0 (0)
			Hypothyroidism	4 (7)	0 (0)
			Adrenal insufficiency	2 (3)	0 (0)
			Hyperthyroidism	1 (1.5)	0 (0)
			Rash	1 (1.5)	0 (0)
			Grade 2 myositis	1 (1.5)	0 (0)
**Herbst et al.**[18]<br>2016<br>Pembrolizumab	Phase 2/3<br>Advanced NSCLC	345 patients on pembrolizumab at dose of 2mg/kg	Severe skin reactions	4 (1)	3(1)
			Colitis	4 (1)	3 (1)
			Hypothyroidism	28(8)	0 (0)
			Hyperthyroidism	12 (4)	0 (0)
			Hypophysitis	1(<1)	1(<1)
			Hepatitis	1(<1)	1(<1)
			Pancreatitis	3 (1)	2 (1)
			Pneumonitis	16(5)	7(2)
		346 patients on pembrolizumab at dose of 10 mg/kg every 2 weeks	Severe skin reactions	7 (2)	6 (2)
			Colitis	4 (1)	3 (1)
			Hypothyroidism	28(8)	0 (0)
			Hyperthyroidism	20 (6)	1 (<1)
			Hypophysitis	2 (1)	1 (<1)
			Hepatitis	2(1)	0 (0)
			Pneumonitis	15(4)	0(0)
**Garon et al.**[48]<br>2015<br>Pembrolizumab	Phase 1<br>Advanced or metastatic NSCLC	495 patients on pembrolizumab at dose of 2 mg/kg or 10 mg/kg every 3 weeks or 10 mg/kg every 2 weeks	Pruritus	53(10.7)	0 (0)
			Rash	48(9.7)	1(0.2)
			Dry skin	18(3.6)	0(0)
			Diarrhea	40 (8.1)	3 (0.6)
			Hypothyroidism	34 (3.6)	1 (0.2)
			↑ ALT	15(6.9)	9(1.8)
			↑ AST	11(3)	3(0.6)
			Pneumonitis	18(3.6)	9(1.8)

**Rosenberg et al.**[50]<br>2016<br>Atezolizumab	Phase 2<br>Locally advanced or metastatic urothelial carcinoma	310 patients on atezolizumab at dose of 1200mg every 3 weeks	Any irAEs<br>Rash<br>↑ ALT<br>↑AST<br>Pneumonitis	23(7)<br>2(1)<br>2(1)<br>2(1)<br>2(1)	15(5)<br>1(<1)<br>1(<1)<br>1(<1)<br>2(1)
**Fehrenbacher et al.**[13]<br>2016<br>Atezolizumab	Phase 2<br>Advanced or metastatic NSCLC	142 patients on atezolizumab at dose of 1200 mg every 3 weeks	Colitis<br>↑ ALT<br>↑ AST<br>Hepatitis<br>Pneumonitis	2 (1)<br>6 (4)<br>6 (4)<br>1 (<1)<br>4 (3)	1 (<1)<br>3 (2)<br>3 (2)<br>0 (0)<br>1 (<1)
**Rittmeyer et al.**[14]<br>2016<br>Atezolizumab	Phase 3<br>Stage IIIB/ IV NSCLC	Safety analysis reported for 609 patients on atezolizumab 1200 mg every 3 weeks	Colitis<br>Hepatitis<br>Pneumonitis	2 (<1)<br>2 (<1)<br>6 (1)	0 (0)<br>2 (<1)<br>0 (0)
**Massard et al.**[12]<br>2016<br>Durvalumab	Phase 1/2<br>Inoperable/ metastatic transitional-cell urothelial carcinoma	61 patients on Durvalumab at dose of 10mg/kg every 2 weeks up to 12 months.	Any irAEs[$]<br>Pruritus<br>Diarrhea<br>Colitis<br>Pneumonitis	14 (23)<br>2 (3.3)<br>6 (9.8)<br>0 (0)<br>0 (0)	3 (4.9)<br>0 (0)<br>0 (0)<br>0 (0)<br>0 (0)

The cumulative numbers for the specific events may be greater than the number reported for organ category because patients with more than one event were counted separately for each event but only once for organ category.
$ Not all immune related adverse events were captured due to limited follow up.
@NSCLC non –small cell lung cancer.
